# PReS-FINAL-2329: Validation of the autoinflammatory activity index (AIDAI)

**DOI:** 10.1186/1546-0096-11-S2-P319

**Published:** 2013-12-05

**Authors:** M Piram, I Kone-Paut, H Lachmann, S Ozen, J Kuemmerle-Deschner, S Stojanov, A Simon, M Finetti, MP Sormani, A Martini, M Gattorno, N Ruperto

**Affiliations:** 1 Pediatrics and Pediatric Rheumatology, CHU de Bicêtre, University of Paris Sud, Le Kremlin Bicêtre, France; 2 National Amyloidosis Centre, Royal Free Campus, University College Medical School, London, UK; 3 Hacettepe University Children's Hospital, Ankara, Turkey; 4 Universitatsklinik fur Kinderheilkunde und Jugendmedizin, Tübingen, Germany; 5 Immunologische Tagesklinik, Haunersches Kinderspital, Muenchen, Germany; 6 Department of General Internal Medicine, Radboud University Medical Center, Nijmegen, Germany; 7 Pediatria II, Reumatologia,PRINTO, Institute Gaslini, Italy; 8 Dipartimento di Scienze della Salute, Università di Genova, Italy; 9 Pediatria II, Reumatologia, PRINTO, Institute Gaslini, Italy; 10 Pediatria II, Reumatologia, PRINTO, Istituto G. Gaslini, Genova, Italy

## Introduction

With the increasing potential for targeted therapies in autoinflammatory diseases, there is the need for validated and standardized assessment tools which can be used to evaluate the level of disease activity and response to therapy. An international collaboration, initiated by Assistance Publique-Hôpitaux de Paris (APHP) in association with the Paediatric Rheumatology International Trials Organization (PRINTO at http://www.printo.it) and supported by the EUROFEVER and EUROTRAPS networks, has previously designed the content and the preliminary scoring of an Auto-Inflammatory Disease Activity Index (AIDAI)

## Objectives

To validate the AIDAI score in the four major hereditary recurrent fever syndromes (HRFs): familial Mediterranean fever (FMF), mevalonate kinase deficiency (MKD), tumor necrosis factor receptor-associated periodic syndrome (TRAPS), and cryopyrin-associated periodic syndromes (CAPS).

## Methods

In 2010, an international collaboration established the content of a disease activity tool for HRFs. Patients completed a one-month prospective diary with 12 yes/no (dichotomous) items prior to a clinical appointment during which their physician assessed their disease activity by a questionnaire. Eight international experts in auto-inflammatory diseases evaluated patient's disease activity by a blinded web-evaluation and a nominal group technique consensus conference with their consensus judgment considered as gold standard. Sensitivity/specificity/accuracy measures and the ability of the score to discriminate active versus inactive patients via the best cut-off score were calculated by a receiver operating characteristic (ROC) analysis.

## Results

Consensus was achieved for 98/106 (92%) cases (39 FMF, 35 CAPS, 14 TRAPS and 10 MKD) with 26 patients declared as having inactive disease and 72 active disease. The median total AIDAI score was14 (range = 0-175). An AIDAI cut-off score ≥ 9 discriminated active versus inactive patients, with sensitivity/specificity/accuracy of 89%/92%/90% respectively and an area under the curve of 98% (95%CI = 96% > 100%).

## Conclusion

The AIDAI score is a valid and simple tool for the assessment of disease activity in FMF/MKD/ TRAPS/CAPS This tool is easy to use in clinical practice and has the potential to be used as a standard efficacy measure in future clinical trials.

## Disclosure of interest

None declared.

